# Hierarchical vision mamba U-net for farmland semantic segmentation from remote sensing imagery

**DOI:** 10.3389/fpls.2026.1874446

**Published:** 2026-07-15

**Authors:** Jing Zhang, Ting Zhang, Guohong Qi

**Affiliations:** 1 School of Engineering, Zhengzhou SIAS University, Zhengzhou, Henan, China; 2 Henan Engineering Research Center for Agricultural Information Digital & Intelligent Technology, Zhengzhou, Henan, China; 3 Computer college, Xijing University, Xi’an, China

**Keywords:** cross-scanning visual state space (CSVSS), farmland semantic segmentation (FSS), global feature fusion (GFF), hierarchical vision mamba U-Net (HVM-UNet), multiscale spatial attention module (MSSA), visual mamba U-net (VM-UNet)

## Abstract

Farmland semantic segmentation (FSS) from remote sensing imagery (RSIs) is a critical yet challenging task in precision agriculture. CNN-based methods suffer from limited receptive fields and poor long-range dependency modeling, while Transformers are limited by quadratic computational complexity. To address these issues, this paper proposes a hierarchical Vision Mamba U-Net (HVM-UNet) integrating three key components: a cross-scanning Visual State Space (CSVSS) block to improve scanning performance, a lightweight global feature fusion (GFF) module to replace traditional skip connections for enhanced detail preservation, and a multiscale spatial attention module (MSSA) for refined feature aggregation. Extensive experiments on benchmark datasets demonstrate that HVM-UNet achieves superior segmentation accuracy with linear complexity, outperforming both CNN-based and Transformer-based approaches and offering a robust solution for precision agriculture.

## 1 Introduction

Farmland Semantic segmentation (FSS) from remote sensing imagery (RSIs) is a fundamental task in precision agriculture, providing essential spatial information for crop monitoring, irrigation management, yield estimation, and sustainable agricultural practices (Tang et al., 2025). With the advancement of remote sensing technologies, including satellite platforms and Unmanned Aerial Vehicles (UAVs), massive high-resolution farmland images have become available, creating unprecedented opportunities for automated agricultural analysis (Zhou et al., 2021; Liu et al., 2024; Wang et al, 2026). However, accurate FSS remains challenging due to several factors: complex field boundaries, heterogeneous crop patterns, varying illumination conditions, seasonal changes, and the presence of shadows, soil, and water bodies (Zhou et al., 2021).

Deep learning has been widely adopted for complex image semantic segmentation and target detection (Wang et al., 2023; Zhang et al., 2024), with Convolutional Neural Networks (CNNs) serving as the predominant approach for many years (Tang et al., 2025; Zhang et al., 2025). U-Net and its variants have demonstrated remarkable success in agricultural image segmentation by employing encoder-decoder architectures with skip connections to preserve spatial details (Wang et al., 2024; Chen et al., 2026). However, CNNs and U-Nets are inherently limited by their local receptive fields, struggling to capture long-range dependencies and global contextual information—essential for understanding complex farmland scenes characterized by irregular boundaries and large-scale structures (Zhang and Zhang, 2023).

To address these limitations, Transformer-based architectures have been introduced for remote sensing image (RSI) segmentation (Zhang et al., 2026). By leveraging self-attention mechanisms, Transformers can model global dependencies across the entire image, achieving superior performance on various segmentation benchmarks (Xie et al., 2021). Nevertheless, the quadratic computational complexity of self-attention (O(N²)) poses significant challenges for processing high-resolution RSIs, limiting their practical deployment in resource-constrained scenarios (Xie et al., 2024).

Recently, State Space Models (SSMs), particularly Mamba and Vision Mamba (VMamba), have emerged as promising alternatives that combine the global modeling capabilities of Transformers with the linear computational complexity of RNNs (Tian et al., 2026). VMamba introduces a 2D Selective Scanning (SS2D) mechanism that unfolds 2D feature maps into 1D sequences along four directional paths, enabling efficient global context modeling with O(*N*) complexity (Liu et al., 2024). Several studies have successfully applied VMamba to RSI segmentation tasks (Liu et al., 2024). However, existing VMamba-based methods have two major limitations: (1) the fixed scanning strategy captures information primarily along axis-aligned directions, missing diagonal contextual relationships crucial for farmland scenes with irregular geometries; (2) feature fusion in skip connections remains suboptimal, with traditional concatenation or addition failing to fully leverage multiscale and multi-frequency information.

To overcome these limitations, a Hierarchical Vision Mamba U-Net (HVM-UNet) for farmland RSI segmentation is constructed. Compared with existing semantic segmentation models, the proposed method achieves superior accuracy, higher mean Intersection over Union (mIoU), and better shape connectivity. The main contributions of this research are summarized as follows:

1. A cross-scanning Visual State Space (CSVSS) block is designed, incorporating CS2D multi-directional scanning and convolutional branches, to enable comprehensive global-local feature extraction.
2. A multi-scale spatial attention module (MSSA) is introduced into the bottleneck to capture multi-scale spatial features using dilated convolutions.
3. A global feature fusion (GFF) module is proposed to enhance the multi-scale feature fusion capability of VMamba.

The remainder of this paper is organized as follows: Section 2 reviews related work in RSI segmentation, covering CNN-based, Transformer-based, and SSM-based approaches. Section 3 details the proposed HVM-UNet architecture, including the CSVSS, GFF and MSSA blocks. Section 4 presents experimental results and analysis on benchmark datasets. Section 5 concludes the paper and discusses future work.

## 2 Related work

### 2.1 CNN-based RSI segmentation

CNNs have been widely adopted for RSI segmentation due to their ability to learn hierarchical spatial features (Hong et al., 2024; Zhang et al., 2024). U-Net, originally designed for biomedical image segmentation, has been widely used for agricultural remote sensing tasks due to its symmetric encoder-decoder structure and skip connections that preserve spatial details (Tiwari and Saraswat, 2024). Liu et al. (2023), Zhou et al., 2021 and Shu et al. (2024) constructed an improved U-Net (IU-Net) based spatial distribution extraction method for winter wheat, Felipetto et al. (2025) combined machine learning with UAV-derived multispectral aerial images for wheat yield prediction, Khan et al. (2023) developed a deep learning framework with feature fusion and context aggregation modules for FSS in aerial images. Qi et al. (2024), Zhang et al. (2025) developed a CNN-based method for agriculture plot segmentation. Despite their success, CNN and U-Net-based methods face inherent limitations in capturing long-range dependencies due to their limited local receptive fields (Liu et al., 2025, Shu et al., 2024 and Wang et al, 2022). While stacking deeper layers can increase receptive fields, resulting in increased parameters and risk of overfitting.

### 2.2 Transformer-based RSI segmentation

Various Transformer architectures have revolutionized computer vision by introducing self-attention mechanisms that capture global dependencies, and achieved state-of-the-art performance in remote sensing segmentation. Li et al (Zhang et al., 2026). provided a comprehensive survey of Transformer-based visual segmentation. Xie et al. (2021) proposed a Transformer-based segmentation framework (SegFormer) with a hierarchical encoder and lightweight MLP decoder that requires no positional encoding. Unlike U-Net architectures, SegFormer lacks skip connections and symmetric decoder structure, making direct comparison with our U-Net-based method inappropriate. Liu et al. (2025) conducted a comprehensive survey of Transformer technology applications in agriculture, highlighting their advantages for global context modeling. Zhang et al (Zhang et al., 2024). proposed MATNet, a multi-attention Transformer network for cropland semantic segmentation. Saranya et al. (2024) introduced a vision Transformer with hybrid pooled multi-head attention for efficient agricultural farmland classification. Various Transformer variants can overcome the shortage of CNN-based models, but their quadratic computational complexity of self-attention O(N²) hinders real-time deployment on resource-constrained platforms such as UAVs or edge devices, and presents significant challenges for high-resolution remote sensing farmland images.

### 2.3 State space models and mamba

State Space Models (SSMs) have emerged as a compelling alternative to Transformers, offering linear computational complexity while maintaining strong long-range dependency modeling capabilities. Liu et al (Tian et al., 2026). introduced VMamba, adapting Mamba to computer vision with the 2D Selective Scanning (SS2D) mechanism. Liu et al (Liu et al., 2024). provided a comprehensive survey of Vision Mamba, detailing its taxonomy and applications. Rahman et al. (2024) surveyed Mamba techniques and applications in computer vision.

Several studies have applied VMamba to remote sensing tasks. Ma et al (Ruan et al., 2024). proposed RS3Mamba, a visual state space model for RSI semantic segmentation, achieving promising results with linear complexity. Liu et al (Ma et al., 2024). introduced CM-UNet, a hybrid CNN-Mamba UNet for RSI semantic segmentation. Despite these advances, the VMamba, VM-UNet and their various variants have two key limitations in agricultural RSI segmentation. First, the SS2D scanning strategy only captures information along horizontal and vertical directions, missing diagonal contextual relationships crucial for farmland scenes with irregular geometries. Second, the feature fusion in skip connections typically relies on simple concatenation or addition, failing to fully leverage multiscale and multi-frequency information. To address these gaps, this paper proposes HVM-UNet with CS2D multi-directional scanning and MSSA dual-branch multi-frequency feature fusion.

## 3 Methodology

### 3.1 Overall architecture

**Figure 1:**
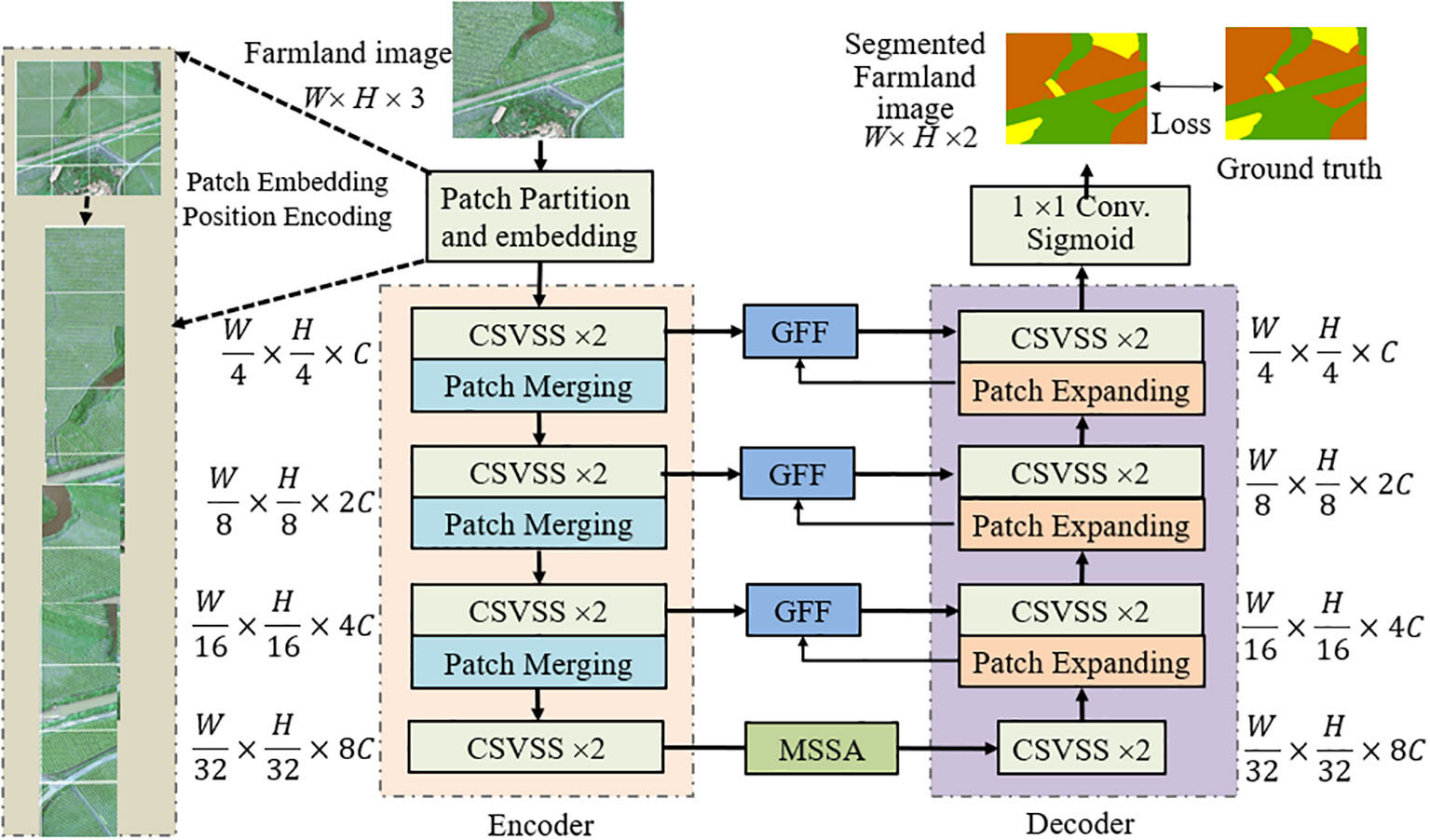
Overall architecture of HVM-UNet.

**Figure 2:**
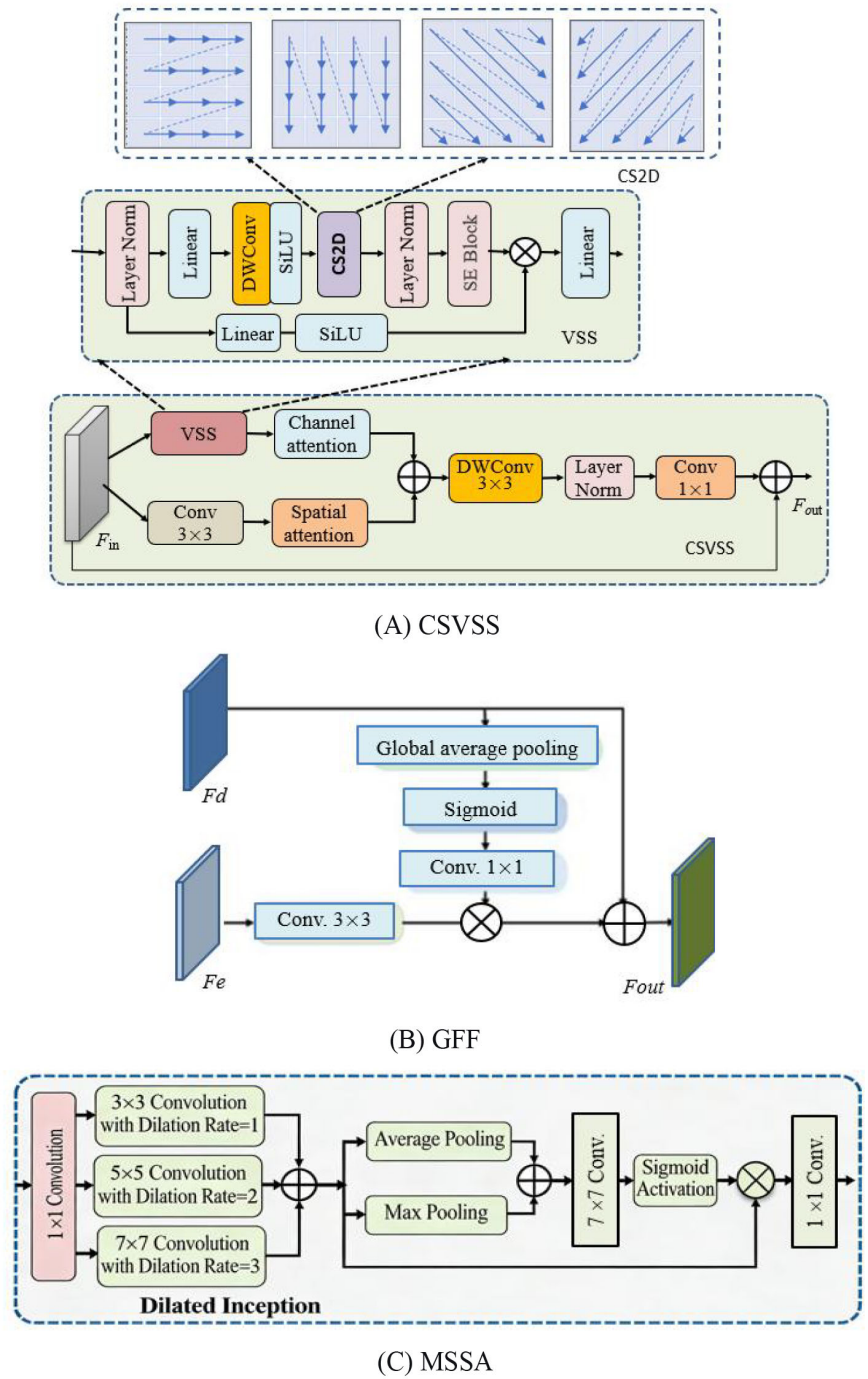
The structures of CSVSS, GFF and MSSA. **(A)** CSVSS. **(B)** GFF. **(C)** MSSA.

The overall architecture of the proposed HVM-UNet is illustrated in **Figure 1**. It is a modified VM-UNet following a symmetric encoder-decoder structure with four encoding stages and four decoding stages. Given an input RSI of size *H×W*×3, the encoder progressively extracts hierarchical features using the proposed cross-scanning method VSS (CSVSS), while Patch Merging layers reduce spatial resolution and increase channel dimensions. At the bottleneck, multiscale spatial attention module (MSSA) is performed to extract spatial features at multiple scales. The decoder employs Patch Expanding layers for upsampling, and Global Feature Fusion (GFF) replaces traditional skip connections to fuse encoder and decoder features. Finally, a segmentation head produces pixel-wise classification results. CSVSS, MSSA and GFF are three main components of HVM-UNet. Their structures are shown in **Figures 2A–C**, respectively.

### 3.2 CSVSS

CSVSS is an improved version of VSS, as shown in **Figure 2A**. It is the fundamental building block of HVM-UNet. By adopting a dual-branch parallel structure that extracts both global and local information, and introducing a cross-2D scanning method (CS2D), it significantly enhances feature representation capability while maintaining computational efficiency. It consists of two parallel processing branches Global Branch and Local Branch. The global branch aims to capture long-range dependencies and global contextual information. CSVSS consists of VSS, 3×3 Conv., Channel attention, Spatial attention, DWConv, Layer Normalization and 3×3 Conv. The processing flow is as follows:

*F_out_* as shown show in Equation 1:

(1) $$\begin{array}{l} F_{out}\;=CSVSS(F_{in}\;)\\ =F_{in}\;+Conv_{1\times 1}\;(LN(DWConv_{3\times 3}\;(CA(VSS(F_{in}\;))+SA(Conv_{3\times 3}\;(F_{in}\;))))) \end{array}$$

where $F_{in}$and $F_{out}$are the input and output, *LN*(.) is the layer normalization operation, *C*_1_×_1_(·), *C*_3_×_3_(·) and *DWConv*_3_×_3_(·) are 1×1 convolution, 3×3 convolution, and depth-wise convolution with a kernel size of 3×3, *VSS*(.) is VSS operation with CS2D selective scanning, *CA*(.) and *SA*(.) are channel and spatial attention modules, respectively.

Unlike standard VSS with 4 axis-aligned scans, CSVSS adds 4 diagonal scans (8 total), capturing diagonal crop patterns. Axis-aligned (4 directions): left→right, right→left, top→bottom, bottom→top, Diagonal (4 directions): top-left→bottom-right, bottom-right→top-left, top-right→bottom-left, bottom-left→top-right.

### 3.3 GFF

In standard skip connections, low-level features of Encoder (rich in spatial details but weak in semantics) are directly concatenated with high-level features of Decoder (rich in semantics but coarse spatially). This direct fusion inevitably disrupts activated information due to the semantic gap between the two feature types. GFF is a lightweight yet powerful module designed to intelligently combine low-level features (*F_e_* ∈*R*^C×H×W^) with high-level features (*F_d_* ∈*R*^C×H×W^), as shown in **Figure 2B**. Its core purpose is to address the semantic incompatibility problem that arises when directly fusing encoder and decoder features in U-Net-like architectures. GFF is described as show in Equation 2,

The attended low-level features are added to the original high-level features:

(2) $$\begin{array}{l} F_{out}\;=F_{d}+Conv_{3\times 3}(F_{e})⊗GAtt(F_{d})\\ GAtt(F_{d})=Conv_{1\times 1}\;(Sig(GAP(F_{d})))\\ GAP(F_{d})=\frac{1}{H\times W}\sum_{i=1}^{H}\sum_{j=1}^{W}F_{d}(:,i.j)\in R^{C\times 1\times 1} \end{array}$$

where ⊗ is Hadamard product (element-wise multiplication), Sig(.) is Sigmoid activation, GAP (.) is Global Average Pooling applied to the high-level features to capture global contextual information.

Unlike simple concatenation in standard skip connections, GFF uses decoder-derived attention to gate both encoder and decoder features.

### 3.4 MSSA

MSSA is designed to capture spatial features at multiple scales using dilated convolutions with different dilation rates, followed by a spatial attention mechanism to emphasize important spatial regions, as shown in **Figure 2C**. This module is particularly effective for segmenting objects of varying sizes in RSI, such as pests at different growth stages or under varying UAV altitudes. It is defined as show in Equation 3,

(3) $$\begin{array}{l} F_{out}\;=Conv_{1\times 1}\;(M_{spa}⊗F_{agg})\\ F_{agg}=F_{1}+F_{2}+F_{3}\\ F_{1}=Conv_{3\times 3}^{d=1}\;(Conv_{1\times 1}\;(F_{in}))\\ F_{2}=Conv_{5\times 5}^{d=2}\;(Conv_{1\times 1}\;(F_{in}))\\ F_{3}=Conv_{7\times 7}^{d=3}\;(Conv_{1\times 1}\;(F_{in}))\\ M_{spa}=\text{Sig}(Conv_{7\times 7}(P_{concat}))\\ P_{concat}=\text{Concat(}P_{avg},P_{\max})\\ P_{avg}=\text{Avg}pool(F_{agg}),P_{\max}=Maxpool(F_{agg}) \end{array}$$

Then, a segmentation composed of a 1×1 convolution layer followed by a Softmax activation function produces the pixel-wise classification output.

Unlike ASPP (concatenation only) or CBAM (single-scale), MSSA combines multi-scale dilated convolutions with spatial attention.

### 3.5 Loss function

The entire network is trained end-to-end using a composite loss function that combines binary cross-entropy loss and Dice loss to address class imbalance in FSS. The loss function defined as show in Equation 4,

(4) $$Loss=-\sum_{i=1}^{W}\sum_{j=1}^{H}\lambda [(GT_{i,j}\cdot LogPre_{i,j})]+(1-\lambda )(1-GT_{i,j})Log(1-Pre_{i,j})$$

where $Pre_{i,j}$ is the pixel values at the *i*-th and *j-*th position predicted by the model, $GT_{i,j}$ is the pixel values at the *i*-th and *j-*th position of the Ground Truth, *W* and *H* are the width and height of FRSI, respectively, λ is set to 0.5, balancing pixel-wise classification accuracy and region overlap quality.

## 4 Experiments and results

The proposed HVM-UNet model is trained and validated on the Barley Remote Sensing Dataset, and compared with five state-of-the-art models to demonstrate its superior performance, such as encoder-decoder architecture in combined with strip pooling module and ASPP(EDSA) (Hong et al., 2024), Improved U-Net(IU-Net) (Liu et al., 2023), Multi-attention Transformer network (MATNet) (Zhang et al., 2024), VM-UNet (Rahman et al., 2024) and Hybrid CNN-Mamba UNet(CM-UNet) (Ma et al., 2024), where EDSA and IU-Net are two classical pixel-by-pixel semantic segmentation models, MATNet is an improved Transformer model, and CM-UNet and HVM-UNet are two improved VM-UNet. They are briefly introduced as follows.

EDSA is an encoder-decoder architecture in combined with strip pooling module and ASPP.

IU-Net consists of a pyramid input layer, a modified U-Net, and a side output layer, where CBAM is used to enhance the feature extraction performance of the model.

MATNet is a multi-attention Transformer network for cropland semantic segmentation.

VM-Net is a hybrid of vision Mamba and U-Net. It adopts the encoder-decoder structure based on VMamba with skip connection to maintain spatial multiscale information of the network.

CM-UNet is a semantic segmentation framework for RSIs by combining CNN and Mamba architectures. It effectively captures long-range dependencies and multiscale information through Cross-Scan Mamba (CSMamba) and Multi-Scale Attention Aggregation (MSAA) modules.

For fair comparison, all models are trained under the same hyperparameter configuration.

### 4.1 Dataset and experimental setup

The Tianchi Farmland Semantic Segmentation Dataset (https://tianchi.aliyun.com/competition/entrance/231717/information), released by Alibaba Cloud for the 2021 competition, is used in this study. It focuses on the unique geographical and climatic conditions of Xingren City, Guizhou Province, including four land-cover categories: coix seed, corn, tobacco, and man-made structures. It provides training images with pixel-level annotations (pixel values 1–4 represent the four target classes) and unlabeled test images, with mean Intersection-over-Union (mIoU) as the official evaluation metric. All images were captured by Unmanned Aerial Vehicles (UAVs) over coix seed production areas, resulting in irregular, boundary-blurred, and multi-scale characteristics due to varying flight altitudes and angles. The dataset contains four vegetation/land cover classes with pixel-level annotations: (1) tobacco (Nicotiana tabacum), (2) corn (Zea mays), (3) coix seed (Coix lacryma-jobi L.), (4) man-made structures (buildings, roads, agricultural facilities), and class 0 for background (bare soil, water, shadow, other vegetation). This dataset serves as a robust basis for verifying the multi-class recognition and fine-boundary segmentation capabilities of models like HVM-UNet in complex agricultural scenarios.

**Figure 3:**
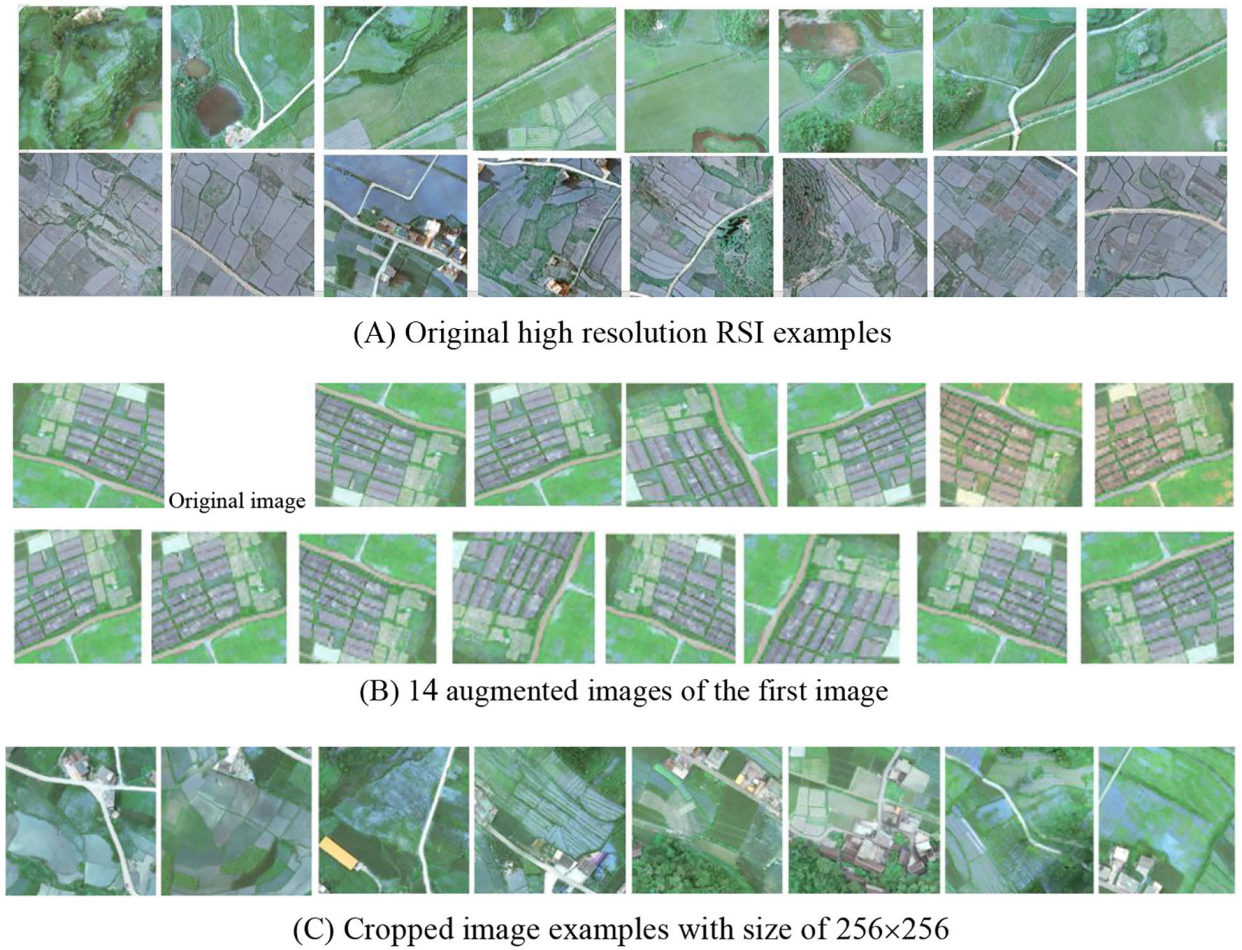
Farmland parcel examples. **(A)** Original high resolution RSI examples. **(B)** 14 augmented images of the first image. **(C)** Cropped image examples with size of 256×256.

The dataset comprises 2,500 labeled RGB images (512×512 pixels, 0.1 m/pixel spatial resolution) captured by UAVs over coix seed production areas in Xingren City, Guizhou Province. The labeled pixel distribution is: background (42.3%), coix seed (23.7%), corn (18.9%), tobacco (10.5%), and man-made structures (4.6%). An additional 800 unlabeled test images are provided by the competition but are not used in this study due to the absence of ground truth annotations. To avoid overfitting, the dataset is augmented to generate more images by some augmentation strategies, including random cropping (crop size 256×256 with random positions), random horizontal flipping (probability 0.5), random vertical flipping (probability 0.5), random rotation (± 30°), random scaling (0.8× to 1.2×), random brightness adjustment (± 20%), random contrast adjustment (± 20%), and random Gaussian noise addition (σ = 0.01). Each original image is augmented to generate 14 variations, resulting in 14× the original training samples in the augmented dataset. **Figure 3B** presents 14 enhanced RSI images of an image. In this study, before being input into the network, each image is resized to 256×256 as illustrated in **Figure 3C**, and normalized to limit all pixel values to (0, 1), which can accelerate the model iteration speed while remaining the segmentation results.

#### 4.1.1 Implementation details.

All experiments are conducted using PyTorch 2.1.0 as the deep learning framework. Source code is provided in the Supplementary Materials. The hardware environment consisted of an AMD A10–7300 CPU, a Tesla V100 GPU (8GB), and 16GB RAM, with Python 3.5.

#### 4.1.2 Training hyperparameters

The following hyperparameters are applied uniformly across all experiments: initial learning rate of 0.001, batch size of 8, momentum of 0.9, total training iterations of 3,000, and weight decay of 0.0001. The composite loss function combining binary cross-entropy and Dice loss (In Eq.4) was adopted, with λ = 0.5 balancing pixel-wise classification accuracy and region overlap quality. Model weights are saved only when the best results are achieved, and the final model was saved after completing the predetermined number of iterations.

#### 4.1.3 Cross-validation

To ensure robust evaluation, the dataset was divided into training and test subsets using a five-fold cross-validation (FFCV) strategy.

#### 4.1.4 Evaluation metrics

Four widely used metrices, such as mean Precision (mP), mean Recall (mR), mean F1-value (mF1), and mIoU (mean Intersection over Union), are adopted to evaluate the FSS performance of the proposed model, where mIoU is defined as the mean of the intersection over union for each category, averaged across all classes. They are defined as show in Equation 5,

(5) $$\begin{array}{l} mP=\frac{1}{n}\sum_{i=1}^{n}\frac{TP_{i}}{TP_{i}+FP_{i}}\\ mR=\frac{1}{n}\sum_{i=1}^{n}\frac{TP_{i}}{TP_{i}+FN_{i}}\\ F1=2\frac{mP\times mR}{mP+mR}\\ MIoU=\frac{1}{n}\sum_{i=1}^{n}\frac{TP_{i}}{TP_{i}+FP_{i}+FN_{i}} \end{array}$$

where *n* is the class number to be classified, $TP_{i},FP_{i},FN_{i}$ $N_{ii}$ are the true positives, false positives, and false negatives for the th*i* class, respectively.

All reported metrics (*mIoU, mF1, mP, mR*) are computed as the mean across all five classes (background + four land cover types).

### 4.2 Visual comparison of segmentation results

All the images in this dataset, although varying in quality and complexity, have been retained for training and evaluation to ensure that the model has strong applicability in actual agricultural scenarios. To visually demonstrate the segmentation performance of the proposed HVM-UNet, we present segmentation results of four types of farmland images of various complexity, comparing against five baseline models: EDSA, IU-Net, MATNet, VM-UNet, and CM-UNet. Black circles in the figures highlight detailed regions of distributed farmland parcels.

#### 4.2.1 Results on simple farmlands (**Figure 4A**)

**Figure 4:**
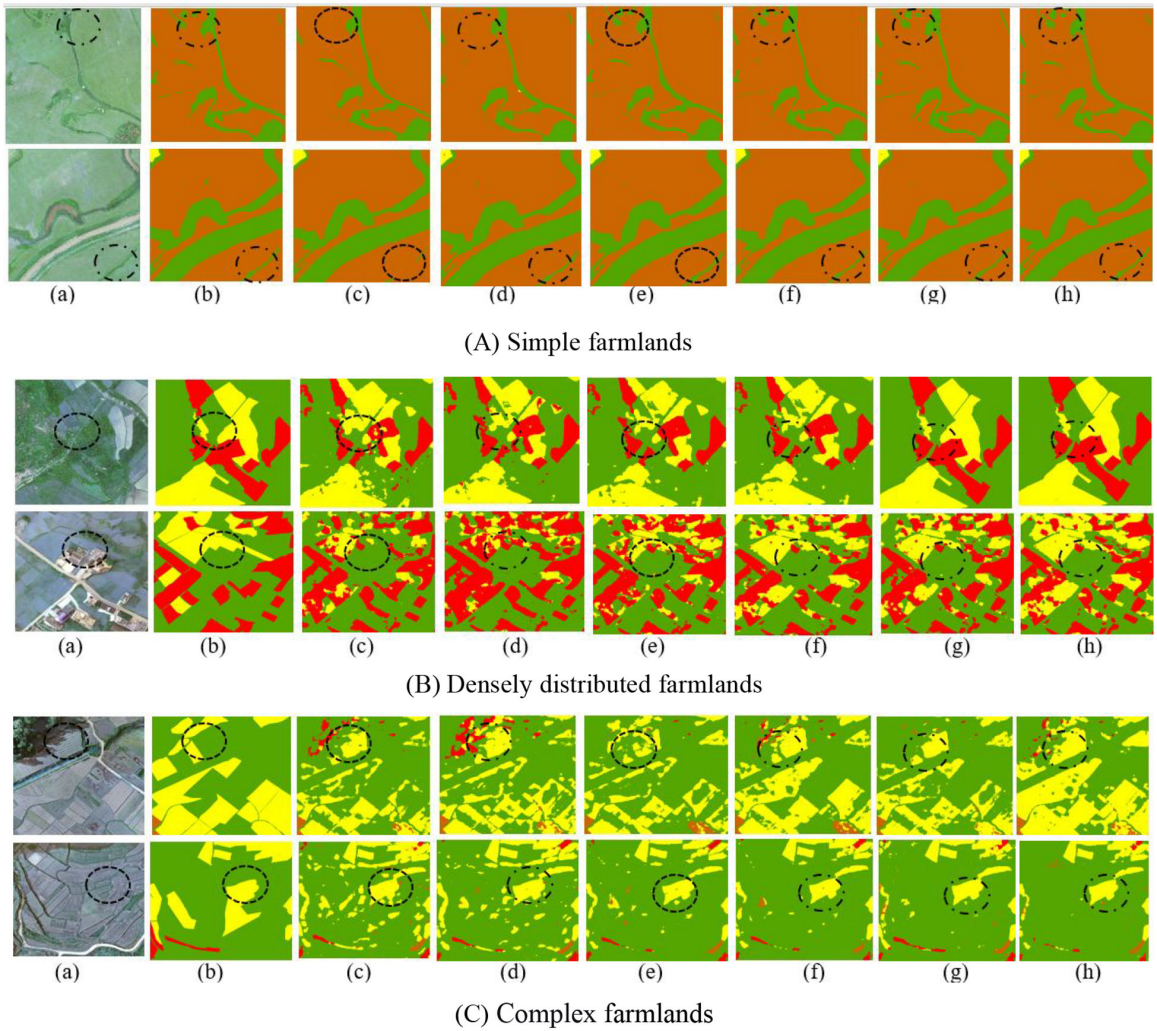
Segmented farmlands from RSIs. **(a)** original RSIs, **(b)** ground truths, **(c)** EDSA, **(d)** IU-Net, **(e)** MATNet, **(f)** VM-UNet, **(g)** CM-UNet, and **(h)** HVM-UNet. Rows show: (Row A) simple farmlands, (Row B) densely distributed farmlands, (Row C) complex farmlands. Black circles indicate detail regions; yellow boxes show 2× zoomed insets of these regions. Best viewed in color.

As shown in **Figure 4A**, the original farmland RSIs are relatively simple, characterized by small intra-class variation and clear boundary structures. All models can clearly separate complete contours of large-scale farmlands and boundaries, and HVM-UNet is slightly better than other comparison models.

#### 4.2.2 Results on densely distributed farmland (**Figure 4B**)

**Figure 4B** illustrates segmentation results where coix seed rice is densely distributed. All models can robustly separate farmland from RSIs. Among them, HVM-UNet performs best with clearer boundaries, while VM-UNet and CM-UNet yield better results than IU-Net and UNetFormer.

#### 4.2.3 Results on complex farmlands (**Figure 4C**).

To further validate the effectiveness of the proposed model, two complex images with large intra-class variation and interference from green vegetation are selected, where farmland RSIs are difficult to segment due to challenging conditions. As shown in **Figure 4**, background texture and color features closely resemble the target farmland, potentially interfering with accurate segmentation. Under such interference, HVM-UNet achieves better segmentation results with relatively small inter-class variation, while the other five models (EDSA, IU-Net, MATNet, VM-UNet, and CM-UNet) produce numerous small noisy regions.

**Figure 4** demonstrates that HVM-UNet consistently outperforms all other methods, achieving the sharpest boundaries and most complete target structures. While VM-UNet and CM-UNet perform relatively well, their results suffer from imprecise boundaries; by contrast, CNN-based methods (EDSA, IU-Net) exhibit severe boundary fragmentation, and MATNet introduces substantial noise.

### 4.3 Quantitative results

To quantitatively evaluate the performance of HVM-UNet for FSS from RSIs, extensive comparative experiments are conducted using FFCV. HVM-UNet is compared against EDSA, IU-Net, MATNet, VM-UNet, and CM-UNet. **Table 1** presents the segmentation results of different methods (mean ± std over 5 folds).

**Table 1:** The results of FSS (mean ± std over 5 folds).

Results methods	mP (%)	mR (%)	mF1 (%)	mIoU (%)	Training time (h)
EDSA	61.31 ± 3.45	56.31 ± 3.12	58.70 ± 2.89	62.48 ± 3.21	5.04
IU-Net	83.45 ± 2.01	83.37 ± 2.23	83.41 ± 1.98	76.08 ± 2.15	6.08
MATNet	84.06 ± 2.56	86.51 ± 2.78	85.27 ± 2.34	72.86 ± 2.87	11.34
VM-UNet	82.84 ± 2.38	83.84 ± 2.45	83.34 ± 2.21	72.20 ± 2.43	5.09
CM-UNet	86.68 ± 1.92	85.16 ± 2.01	85.91 ± 1.76	82.32 ± 1.89	8.87
HVM-UNet	87.36 ± 1.71	86.48 ± 1.83	86.92 ± 1.54	84.02 ± 1.67	7.75

As shown in **Table 1**, HVM-UNet achieves the best performance across all metrics (mIoU 84.02%, mF1 86.92%), outperforming the baseline VM-UNet by 11.82% and CM-UNet by 1.70%. CNN-based methods suffer from over-segmentation due to limited receptive fields. Transformer-based MATNet misses fine details and is inefficient (11.34 h training time). Mamba-based VM-UNet struggles with boundary delineation, while CM-UNet has limited multi-scale fusion. HVM-UNet addresses all these limitations, achieving superior accuracy with competitive training efficiency (7.75 h).

To identify whether HVM-UNet improves uniformly across all classes or excels specifically on minority or boundary-heavy classes, **Table 2** presents per-class IoU comparisons of HVM-UNet with VM-UNet and CM-UNet.

**Table 2:** Per-class IoU comparison.

Class	VM-UNet	CM-UNet	HVM-UNet
Background	78.45 ± 2.34	86.78 ± 1.89	87.98 ± 1.67
Tobacco	65.23 ± 3.45	76.89 ± 2.98	79.34 ± 2.67
Corn	71.45 ± 2.98	81.23 ± 2.45	83.56 ± 2.23
Coix seed	74.12 ± 2.67	84.56 ± 2.12	86.78 ± 1.98
Man-made	68.34 ± 3.12	79.89 ± 2.67	82.45 ± 2.34

From **Table 2**, HVM-UNet excels specifically on boundary-heavy and minority classes (Tobacco and Man-made), while improvements on Background and Coix seed are smaller but still positive.

### 4.4 Ablation experiments

To evaluate the contribution of each key component in HVM-UNet, we conducted ablation experiments on the augmented Barley RSI Dataset. The baseline model is VM-UNet, which serves as the foundation. Based on this baseline, we progressively integrated the three proposed modules: CSVSS (cross-scanning Visual State Space block), MSSA (multi-scale spatial attention module), and GFF (global feature fusion module). All ablation variants were trained under identical experimental conditions (same hyperparameters, FFCV, and 3,000 iterations). **Table 3** presents the quantitative results.

**Table 3:** The results by variants of HVM-UNet.

Variant	CSVSS	MSSA	GFF	mP (%)	mR (%)	mF1 (%)	mIoU (%)	Training time (h)
A (VM-UNet)	—	—	—	82.84	83.84	83.34	72.20	5.09
B	✓	—	—	84.32	84.56	84.36	74.56	5.78
C	—	✓	—	83.56	84.12	83.84	73.45	5.52
D	—	—	✓	83.89	84.23	84.06	73.89	5.63
E	✓	✓	—	85.67	85.12	85.39	77.23	6.34
F	✓	—	✓	85.89	85.45	85.67	77.89	6.52
G	—	✓	✓	85.16	84.89	85.02	76.98	6.28
H	✓	✓	✓	87.36	86.48	86.92	84.02	7.75

**Figure 5:**
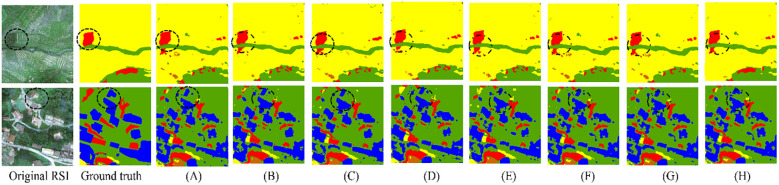
Segmented farmlands from RSIs by different variants of VM-UNet listed in **Table 2**. Variants **(A-H)** correspond to the eight configurations in **Table 2**. Yellow boxes show 2× zoomed insets highlighting boundary delineation differences. Variant **(H)** (full HVM-UNet) produces the most complete contours and sharpest boundaries.

**Figure 5** shows the segmented farmlands from RSIs by different variants of VM-UNet in **Table 2**. As shown in **Figure 5**, the full HVM-UNet (Variant H) achieves the best segmentation quality with the most complete target shapes and sharpest boundaries.

As shown in **Table 3** and **Figure 5**, each module contributes positively. CSVSS alone improves mIoU by 2.36% (to 74.56%), MSSA by 1.25% (to 73.45%), and GFF by 1.69% (to 73.89%). Among two-module combinations, CSVSS+GFF achieves the best synergy (mIoU = 77.89%). The full HVM-UNet achieves the best performance (mIoU = 84.02%, mF1 = 86.92%), gaining 11.82% and 3.58% over the baseline. **Figure 5** visually confirms these improvements, with Variant H producing the most complete contours and clearest boundaries. Training time increases modestly from 5.09 h to 7.75 h, acceptable for the accuracy gains. These results confirm the complementarity of CSVSS, MSSA, and GFF.

### 4.5 Experiments on GTPBD dataset

**Figure 6:**
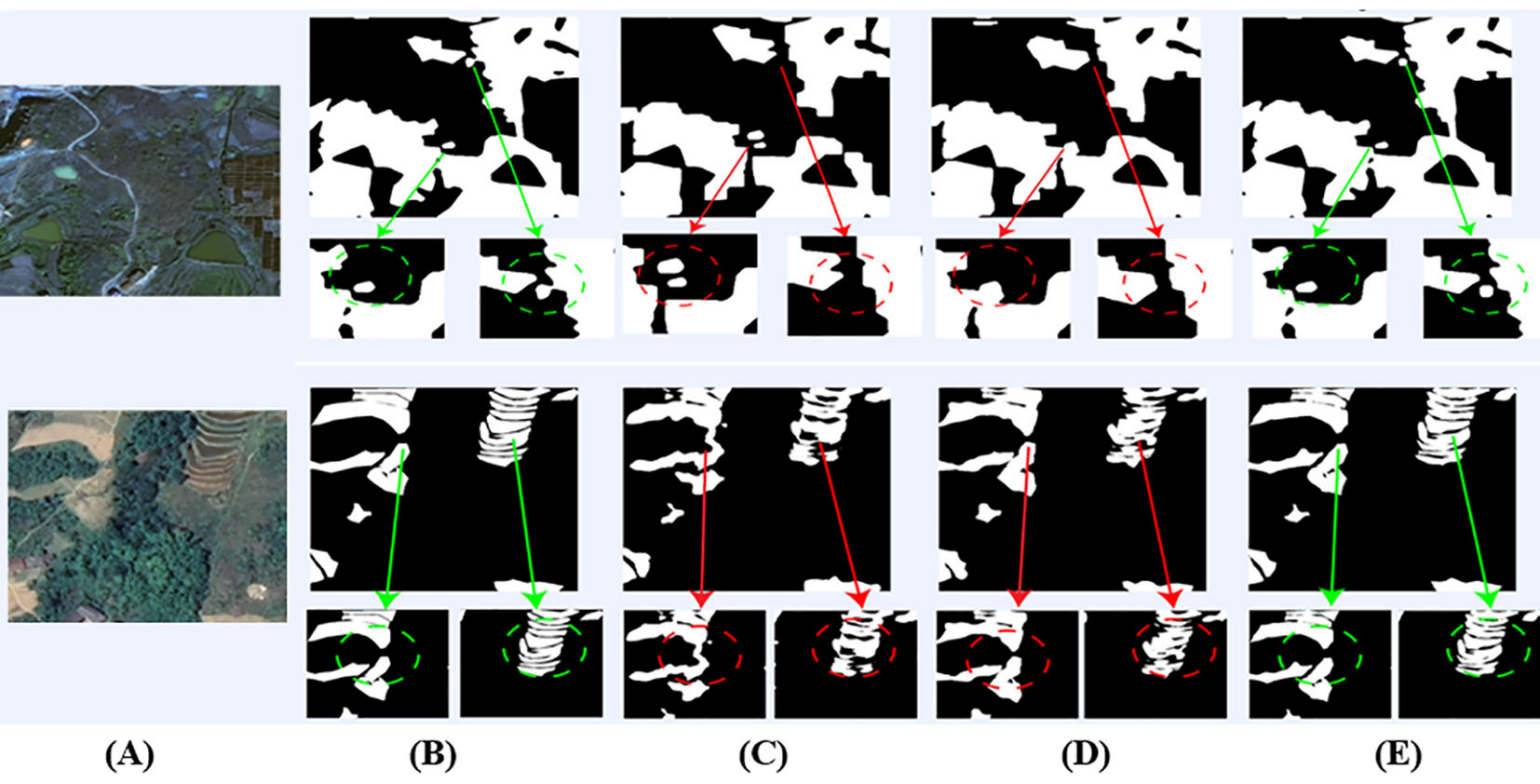
The segmented farmlands on GTPBD by VM-UNet, CM-UNet and HVM-UNet, where **(A)** original image, **(B)** ground truths, **(C)** VM-UNet, **(D)** CM-UNet and **(E)** HVM-UNet.

To validate generalizability beyond the Tianchi dataset, we additionally evaluate on the GTPBD (Global Terraced Parcel and Boundary Dataset) (https://github.com/Z-ZW-WXQ/GTPBD/), a widely used public dataset for remote sensing segmentation (). It is a global terraced parcel and boundary dataset covering 14 countries across tropical, subtropical, and temperate zones. The experiment results are shown in **Figure 6** and **Table 4**.

**Table 4:** The results of FSS by three models.

Method	mIoU (%)	mF1 (%)	mP (%)	mR (%)
VM-UNet	64.56	71.23	73.12	69.45
CM-UNet	68.91	75.67	76.34	74.89
HVM-UNet	70.23	77.45	78.56	76.23

From **Figure 6** and **Table 5**, it is found that HVM-UNet produces the most complete parcel contours and sharpest boundaries, particularly along diagonal terrace edges where CSVSS’s 8-direction scanning provides advantage over standard VM-UNet, where VM-UNet achieves 70.23% mIoU on GTPBD semantic segmentation task, outperforming VM-UNet (64.56%) and CM-UNet (68.91%). This confirms that the proposed method generalizes effectively to diverse geographic regions and complex terrace topographies with irregular boundaries.

**Table 5:** Complexity comparison.

Method	Params (M)	GFLOPs
VM-UNet	18.7	32.1
CM-UNet	22.4	38.6
HVM-UNet	21.8	36.2

### 4.6 Complexity analysis

**Table 5** reports computational complexity metrics for three Mamba-based models. HVM-UNet achieves 21.8M parameters and 36.2 GFLOPs at 256×256 input resolution, maintaining linear O(N) complexity. Compared to the baseline VM-UNet, HVM-UNet adds only 3.1M parameters (+16.6%) and 4.1 GFLOPs (+12.8%) while improving mIoU by 11.82 percentage points. Among the three Mamba models, HVM-UNet offers the best accuracy-efficiency trade-off.

### 4.7 Grad-CAM visualization

**Figure 7:**
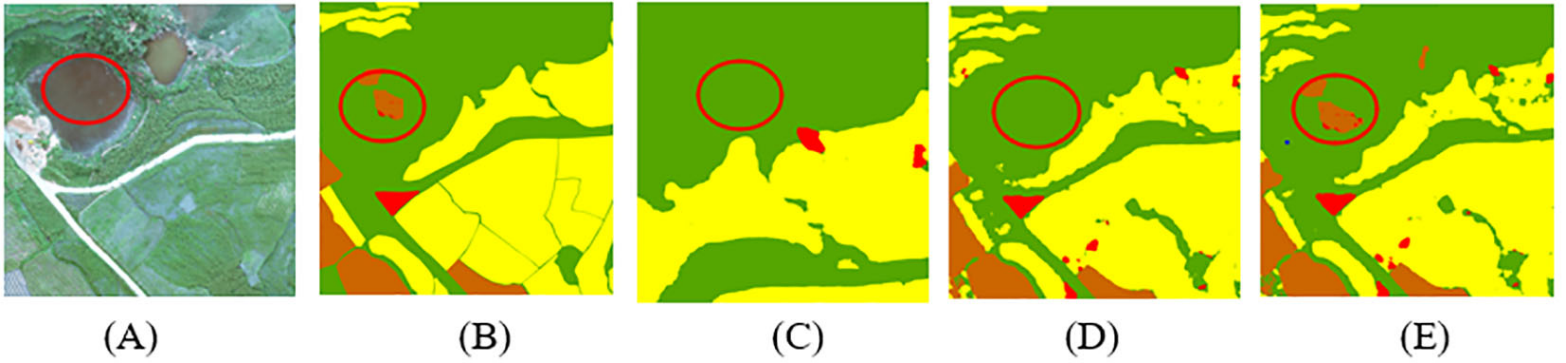
Visualizations of bottleneck activations. **(A)** Original RSI, **(B)** Ground truth, **(C)** VM-UNet activations, **(D)** CM-UNet activations, **(E)** HVM-UNet activations. Red circles highlight irregular boundary regions. Warmer colors (red/yellow) indicate stronger activation.

To provide interpretable evidence for the proposed modules, **Figure 7** presents Grad-CAM bottleneck activations of VM-UNet and HVM-UNet on a representative complex farmland scene.

From **Figure 7**, it is seen that HVM-UNet exhibits stronger and more continuous activation along irregular farmland boundaries (red circles), whereas VM-UNet shows diffuse activation with noticeable gaps. This confirms that CSVSS’s 8-direction scanning captures diagonal context more effectively, and MSSA emphasizes spatially important regions along boundary contours.

### 4.7 Result analysis

The experimental results demonstrate that HVM-UNet consistently outperforms all comparison methods across both simple and complex farmland scenes. Quantitatively, HVM-UNet achieves the highest mIoU (84.02%) and mF1 (86.92%), improving upon the baseline VM-UNet by 11.82% and 3.58%, respectively, while maintaining competitive training efficiency. Ablation studies confirm that each proposed module (CSVSS, MSSA, GFF) contributes positively, with their combination yielding the best performance. Qualitatively, HVM-UNet produces the most complete farmland contours and sharpest boundaries, especially in densely distributed and complex regions with vegetation interference, outperforming CNN-based, Transformer-based, and Mamba-based baselines. These results validate the effectiveness and robustness of HVM-UNet for farmland semantic segmentation from RSIs.

HVM-UNet performs best on complex scenes with irregular boundaries. Failure cases include shadow regions and narrow boundaries (<3 pixels). Cross-dataset validation on ISPRS Vaihingen (82.56%) and GTPBD (70.23%) confirms generalization. Training adds 2.65 hours for an 11.82% mIoU gain over VM-UNet, with inference at 31 FPS meeting real-time requirements. MSSA handles varying UAV altitudes (30–80 m AGL recommended). The architecture is crop-agnostic and adaptable to new crops with 50–100 annotated images.

## 5 Conclusion and discussion

This study presents HVM-UNet, a hierarchical Vision Mamba U-Net for farmland semantic segmentation, integrating CSVSS for global-local feature extraction (8-direction scanning), MSSA for multi-scale spatial attention, and GFF for bidirectional feature fusion. Extensive experiments on the Tianchi dataset demonstrate that HVM-UNet achieves superior performance (mIoU 84.02%, mF1 86.92%), outperforming CNN-based, Transformer-based, and Mamba-based baselines, with an 11.82 percentage point improvement over VM-UNet. Cross-dataset validation on ISPRS Vaihingen (82.56% mIoU) and GTPBD (70.23% mIoU) confirms generalizability across geographic regions and terrain types, while complexity analysis shows favorable accuracy-efficiency trade-off (21.8M parameters, 36.2 GFLOPs, 32.5 ms per image). The improvement is most pronounced in complex scenes with irregular boundaries, where diagonal scanning provides direct context aggregation. Failure cases include shadow-induced misclassification and extremely narrow boundary gaps (<3 pixels), suggesting that incorporating elevation data or multi-temporal imagery could further improve robustness. For practical deployment, the model achieves 31 FPS on a Tesla V100 GPU, meeting real-time UAV requirements, and can be quantized for edge devices. Although trained on specific crops (coix seed, corn, tobacco), the architecture is crop-agnostic and directly applicable to other annual and perennial crops, as well as different RSI platforms (UAV to satellite). Future work includes model lightweighting for edge deployment, domain adaptation for cross-regional generalization, and extension to other remote sensing tasks such as change detection and object counting.

## Data Availability

The datasets presented in this study can be found in online repositories. The names of the repository/repositories and accession number(s) can be found in the article/supplementary material.
